# Association of serum vitamin D with anxiety in US adults: a cross-sectional study

**DOI:** 10.3389/fnut.2024.1371170

**Published:** 2024-03-14

**Authors:** Zongliang Wen, Long Bai, Shenqin Wu, Jialin Chen, Hamdi Abdirizak Jama, Joyce D. Sawmadal

**Affiliations:** ^1^School of Management, Xuzhou Medical University, Xuzhou, China; ^2^School of Public Health, Xuzhou Medical University, Xuzhou, China; ^3^Affiliated Hospital of Xuzhou Medical University, Xuzhou, China

**Keywords:** serum vitamin D, anxiety, NHANES, smoothed curve fitting, weighted logistic regression

## Abstract

**Objective:**

There have been proposals that vitamin D may be associated with a reduction in the incidence of anxiety disorders. However, the findings thus far have been inconsistent, warranting further investigation. The purpose of this paper is to explore the link between serum vitamin D and anxiety.

**Methods:**

Data are from the National Health and Nutrition Examination Survey (NHANES) in the United States from 2007 to 2012. Study included a total of 12,232 participants, and through the multivariate logistic regression to study the relationship between serum vitamin D and anxiety, smooth curve fitting is used to study the nonlinear relationship between serum vitamin D levels and anxiety.

**Results:**

Serum vitamin D levels demonstrated a negative correlation with anxiety (*p* < 0.001). Vitamin D exhibited a significant impact on anxiety (Q4:OR = 0.774, 95% CI: 0.663–0.903, *p* < 0.01), and this effect remained significant even after adjusting for confounding variables (Q4:OR = 0.781, 95% CI: 0.669–0.912, *p* < 0.01). Smoothed curve fitting revealed a negative association between serum vitamin D levels and the risk of anxiety, and these findings persisted after accounting for confounding variables.

**Conclusion:**

Serum vitamin D levels were inversely associated with anxiety risk in US adults. In the future, more accurate prospective studies are needed to confirm this result.

## 1 Introduction

Anxiety is a common mental illness, mental health in the patient’s quality of life and had a profound influence on overall health (1). Anxiety disorders are a devastating and very common clinical condition that is one of the major causes of global disability (2). These disorders are often accompanied by emotional instability, cognitive dysfunction, and social problems, placing a heavy burden on the patient and the family (3, 4). In order to better understand and treat these mental disorders, researchers have been searching for factors that may be related to the onset of mental impairment and the severity of symptoms.

Vitamin D is a fat-soluble vitamin that is critical not only for bone health, but also for immune system and nervous system function (5–7). In one study, 5.9% of Americans were considered to be vitamin D deficient (serum vitamin D concentration less than 30 mmol or 12 ng/mL), and 24% of Americans were considered to be vitamin D insufficient (serum vitamin D concentration less than 50 mmol or less than 20 ng/mL) (8). Recent studies have shown that vitamin D deficiency is associated with a variety of health problems, including cardiovascular disease, autoimmune disease, and neuropsychiatric disorders (9–11). Vitamin D is therefore not only an essential factor in the maintenance of physical health, but may also have a significant role to play in the onset and progression of mental health problems.

However, there is still some degree of uncertainty about the association of vitamin D with anxiety disorders. A cohort study from the National FondaMental Expertise Centre (FACE-SZ) found that vitamin D supplementation reduces symptoms of depression and anxiety, but treatment of patients with vitamin D deficiency remains inadequate (12), other studies did not find a significant association, for example, in a related study evaluating the relationship between serum vitamin D and anxiety in adult cancer patients before and after chemotherapy, no correlation was found between anxiety scores and serum vitamin D levels in either group (*p* > 0.05). In another study on vitamin D and mental health in pregnant adolescents in Iran, there was no statistically significant association between vitamin D and anxiety (*p* > 0.05) (13, 14).

Therefore, this study was to explore the relationship between serum vitamin D levels and anxiety and its internal mechanism. By gaining insight into these associations, we can better understand these common mental disorders and provide more information and possibilities for their prevention and treatment. These findings are expected to have a positive impact on clinical practice and public health policies in the field of mental health.

## 2 Methods

### 2.1 Study population

**Figure 1:**
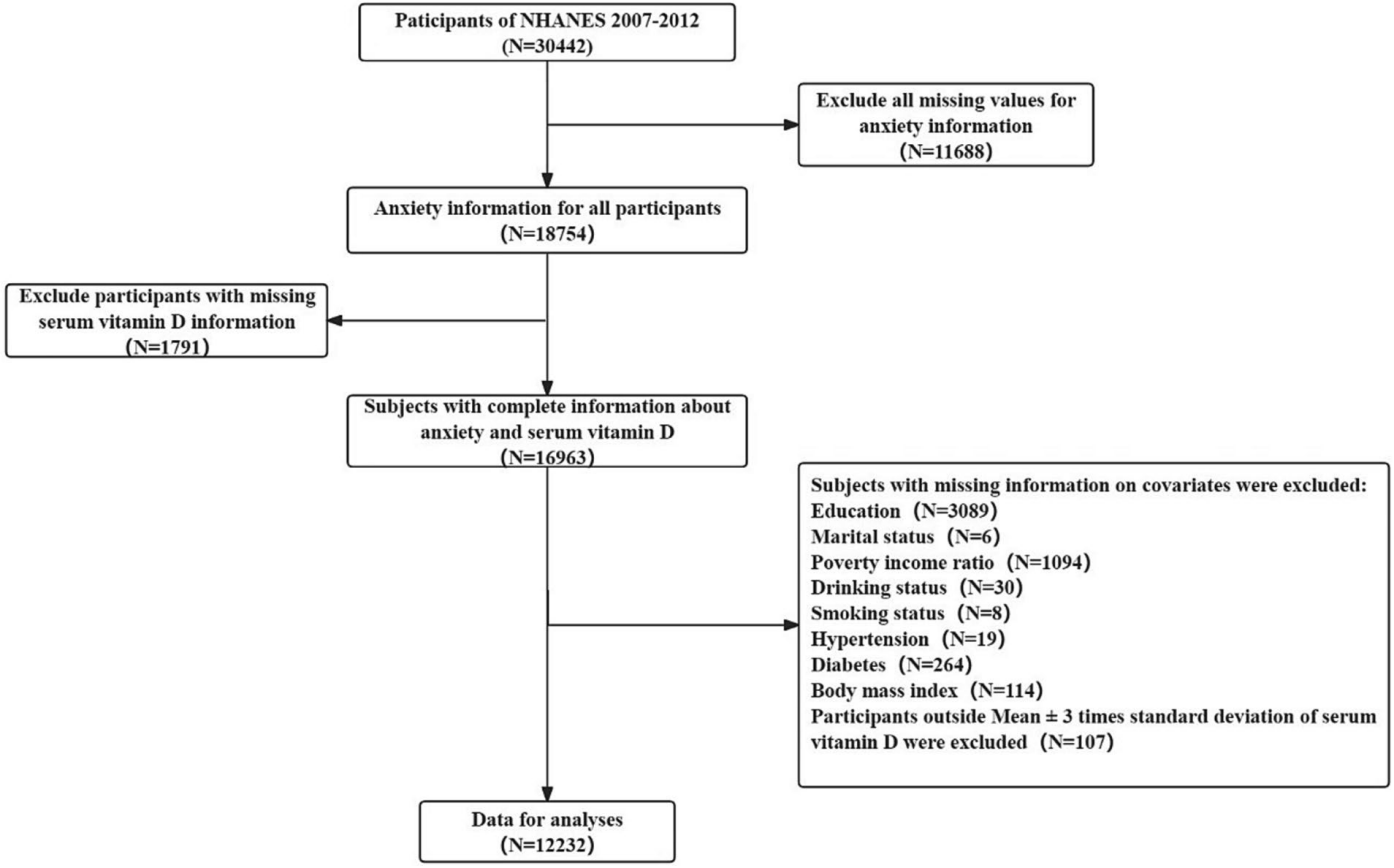
Flow chart for participants recruitment of this study, NHANES 2007–2012.

The National Health and Nutrition Examination Survey (NHANES) is a research program designed to assess the health and nutritional status of adults and children in the United States. It conducts a series of surveys on different populations or health topics. The survey is unique in that it combines interviews and physical examinations. In 1999, the survey became an ongoing program covering a wide range of health and nutrition measures. The project samples about 5,000 people in counties across the country, with 15 counties visited each year. Random selection is made through a statistical process using the United States Census information. Three cycles of data from 2007–2012 were used for this cross-sectional study. Participants with incomplete information on anxiety, missing serum vitamin D data and basic information, and age < 20 years were excluded. In addition, subjects with missing serum vitamin D information and subjects with mean ± standard deviation more than three times the mean were also excluded. Finally, a total of 12,232 individuals were included in the final analysis (Figure 1).

### 2.2 Measurement of serum vitamin D concentrations

The principle of the assay is the quantitative determination of 25-hydroxyvitamin D3 (25OHD3), epi-25-hydroxyvitamin D3 (epi-25OHD3) and 25-hydroxyvitamin D2 (25OHD2) in human serum using ultra-high performance liquid chromatography–tandem mass spectrometry (UHPLC–MS/MS). To process serum samples, an ethanol solution containing the three internal standards and 69–72% methanol solution is added first, followed by hexane. Analytes from the water phase extraction to n-hexane layer (liquid–liquid extraction), and then drying under vacuum. Extract with 69–72% of methanol to dissolve. To extract uniform fluid injection PFP column,25OHD3, epi-25OHD3, 25OHD2 and internal standard (is) 26,27-hexadeuterium-25-hydroxyvitamin D3, 6,19,19-trideuterium-3-epi-25-hydroxyvitamin were isolated D3 and 6,19, 19-Trideuterium-25-hydroxyvitamin D2. Use MS–ms in Thermo TSQ Vantage system, using positive ion mode of atmospheric pressure chemical ionization. Quantitative is by comparing the unknown peak area of analytes and calibrator known quantity in the solution of the analyte peak area. According to the internal standard peak area of objects with unknown calibration fluid matching of internal standard peak area in comparison, the calculation results are correct.

In this study, “serum vitamin D” refers to the concentration of 25OHD2 + 25OHD3 in the blood (nmol/L). Measurement of serum levels of 25OHD2 and 25OHD3 can provide insight into whether an individual has enough of the precursor form in their body for further metabolism to the active form in response to vitamin D intake and sun exposure. This provides an assessment of an individual’s overall vitamin D status and can help guide vitamin D supplementation or adjustments to diet and sun exposure.

### 2.3 Outcome variable

In NHANES 2007–2012, information on anxiety status was collected by questionnaire using a computer assisted personal interview (interviewer administration) system during physical examination at a mobile examination center (MEC). Anxiety was evaluated during the individual interview by the following question, “During the past 30 days, about how many days did you feel worried, nervous, or anxious?” This evaluation is based on the 14-item Healthy Days measure developed by the United States Centers for Disease Control and Prevention (CDC), which has been incorporated into the Health-Related Quality of Life (HRQoL) assessment. The reliability of HRQoL monitoring questions is moderate to excellent (15). Anxiety states were categorized as “no” (feeling anxious 0 to 6 days per month) and “yes” (feeling anxious 7 to 30 days per month) (16).

### 2.4 Covariates

Demographic information was collected using a standardized questionnaire, including gender (male or female), age, race (Mexican American, other Hispanic, non-Hispanic white, non-Hispanic black, or other race), marital status (not living alone, living alone), education (< high school, high school, > high school), and poverty income ratio. Other confounding variables include smoking (defined as smoking at least 100 cigarettes in a lifetime) and alcohol consumption (defined as drinking at least 12 drinks in a year) (17). Hypertension and diabetes were derived from self-reported physician diagnosis (yes/no). Body mass index is calculated weight (kg) divided by the square of height (m^2^).

### 2.5 Statistical analysis

The baseline information was expressed as mean ± standard deviation (SD) for continuous variables and as frequency (composition ratio) [*n*(%)] for categorical variables. For continuous variables, Kruskal Wallis rank sum tests were performed and Fisher’s exact probability tests were performed for count variables with theoretical counts less than 10. Multivariate logistic regression used appropriate weights. Smoothed curve fitting was employed to explore the non-linear association of serum vitamin D with anxiety symptoms. Model 1 was unadjusted, model 2 was adjusted for gender, age, and race, and model 3 was adjusted for all variables including gender, age, race, education, marital status, body mass index, poverty income ratio, drinking status, smoking status, diabetes, and hypertension. In the regression model, each quantile median serum vitamin D levels are used for continuous variables, to calculate the *p* for trend. *p* values <0.05 on both sides were considered statistically significant. We merged and transformed the data using SPSS 27.0, followed by weighted analyses and smoothed curve fitting using EmpowerStats software.^1^

## 3 Results

The object of study for a total of 12,232 people. Table 1 shows the characteristics of a stratified study population based on serum vitamin D quartiles from the National Health and Nutrition Examination Survey (NHANES) 2007–2012. The analyses revealed significant differences among serum vitamin D levels and various factors. In demographic characteristics, was significantly associated with age and serum vitamin D levels (*p* < 0.001). Significant differences (*p* < 0.001) were found between other variables such as gender, race, education, marital status, smoking status, drinking status, hypertension, body mass index, poverty income ratio, and serum vitamin D.

**Table 1:** Study population characteristics based on the serum vitamin D group in NHANES, 2007–2012 (*N* = 12,232).

Variables	Q1	Q2	Q3	Q4	*p* value
N	3,059	3,074	3,046	3,053	
Age	45.90 ± 17.18	47.43 ± 17.30	49.98 ± 17.46	54.06 ± 17.97	<0.001
Body mass index	30.81 ± 8.12	29.65 ± 6.50	28.50 ± 6.04	27.45 ± 5.77	<0.001
Poverty income ratio	2.20 ± 1.54	2.36 ± 1.60	2.61 ± 1.65	2.84 ± 1.67	<0.001
Gender, %					<0.001
Male	1,408(46.03)	1,684(54.78)	1,654(54.30)	1,361(44.58)	
Female	1,651(53.97)	1,390(45.22)	1,392(45.70)	1,692(55.42)	
Race, %					<0.001
Mexican American	573(18.73)	629(20.46)	430(14.12)	184(6.03)	
Other Hispanic	289(9.45)	411(13.37)	323(10.60)	202(6.62)	
Non-Hispanic White	623(20.37)	1,183(38.48)	1715(56.30)	2,245(73.53)	
Non-Hispanic Black	1,281(41.88)	568(18.48)	362(11.88)	260(8.52)	
Other Races	293(9.58)	283(9.21)	216(7.09)	162(5.31)	
Educational level					<0.001
<High school	876(28.64)	889(28.92)	775(25.44)	626(20.50)	
High school	743(24.29)	666(21.67)	676(22.19)	702(22.99)	
>High school	1,440(47.07)	1,519(49.41)	1,595(52.36)	1725(56.50)	
Marital status					<0.001
Not living alone	1,568(51.26)	1840(59.86)	1944(63.82)	1946(63.74)	
Living alone	1,491(48.74)	1,234(40.14)	1,102(36.18)	1,107(36.26)	
Smoking status					<0.001
Smoker	1,343(43.90)	1,340(43.59)	1,445(47.44)	1,478(48.41)	
Never smoker	1716(56.10)	1734(56.41)	1,601(52.56)	1,575(51.59)	
Drinking status					
Yes	2097(68.55)	2,203(71.67)	2,320(76.17)	2,323(76.09)	<0.001
No	962(31.45)	871(28.33)	726(23.83)	730(23.91)	
Diabetes					0.059
Yes	415(13.57)	395(12.85)	351(11.52)	361(11.82)	
No	2,644(86.43)	2,679(87.15)	2,695(88.48)	2,692(88.18)	
Hypertension					<0.001
Yes	1,077(35.21)	1,009(32.82)	1,042(34.21)	1,196(39.17)	
No	1982(64.79)	2065(67.18)	2004(65.79)	1857(60.83)	
Anxiety					0.004
No	2,227(72.80)	2,243(72.97)	2,276(74.72)	2,331(76.35)	
Yes	832(27.20)	831(27.03)	770(25.28)	722(23.65)	

Continuous variable: mean ± standard deviation; serum vitamin D was classified according to quartiles, Q1 to Q4 (Q1 ≤ 44.10 nmol/L; 44.10 nmol/L < Q2 ≤ 61.10 nmol/L; 61.10 nmol/L < Q3 ≤ 78.60 nmol/L; Q4 > 78.60 nmol/L). *p* value: for continuous variables, Kruskal Wallis rank sum tests were performed and Fisher’s exact probability tests were performed for count variables with theoretical counts less than 10.

Table 2 shows the characteristics of the participants according to the number of days of anxiety. A total of 3,155 participants engaged in anxious behavior for 7 or more days in a 30-day period. The mean age of anxious subjects was 46.70 ± 16.22 years, which was significantly lower than the age of non-anxious subjects (50.25 ± 18.16 years, *p* < 0.001). It was also found that anxious subjects had higher body mass index levels than non-anxious subjects and lower serum vitamin D levels than non-anxious subjects (60.89 ± 24.14 nmol/L, *p* < 0.001). Females were more prone to anxiety (59.46%). Significant differences were found between the two groups in gender, age, body mass index, poverty income ratio, serum vitamin D, race, education, marital status, smoking status, drinking status, hypertension, and diabetes (*p* < 0.05).

**Table 2:** Characteristics of participants classified according to the number of days of anxiety.

variables	<7	≥7	*p* value
N	9,077	3,155	
Age	50.25 ± 18.16	46.70 ± 16.22	<0.001
Body mass index	28.91 ± 6.57	29.67 ± 7.35	<0.001
Poverty income ratio	2.60 ± 1.64	2.21 ± 1.59	<0.001
Serum vitamin D	63.23 ± 25.03	60.89 ± 24.14	<0.001
Gender			<0.001
Male	4,828(53.19)	1,279(40.54)	
Female	4,249(46.81)	1876(59.46)	
Race			<0.001
Mexican American	1,360(14.98)	456(14.45)	
Other Hispanic	860(9.47)	365(11.57)	
Non-Hispanic White	4,204(46.31)	1,562(49.51)	
Non-Hispanic Black	1885(20.77)	586(18.57)	
Other Races	768(8.46)	186(5.90)	
Educational level			<0.001
<High school	2,263(24.93)	903(28.62)	
High school	2,119(23.34)	668(21.17)	
>High school	4,695(51.72)	1,584(50.21)	
Marital status			<0.001
Not living alone	5,540(61.03)	1758(55.72)	
Living alone	3,537(38.97)	1,397(44.28)	
Smoking status			<0.001
Smoker	4,004(44.11)	1,602(50.78)	
Never smoker	5,073(55.89)	1,553(49.22)	
Drinking status			
Yes	6,580(72.49)	2,363(74.90)	0.009
No	2,497(27.51)	792(25.10)	
Diabetes			0.003
Yes	1,082(11.92)	440(13.95)	
No	7,995(88.08)	2,715(86.05)	
Hypertension			0.028
Yes	3,158(34.79)	1,166(36.96)	
No	5,919(65.21)	1989(63.04)	

Continuous variable: mean ± standard deviation. *p* value: for continuous variables, Kruskal Wallis rank sum tests were performed and Fisher’s exact probability tests were performed for count variables with theoretical counts less than 10.

In Table 3, logistic regression analyses were performed between each variable and anxiety. We found that females had a higher risk of anxiety compared to males (OR = 1.506, 95% CI: 1.349–1.681, *p* < 0.001), older people aged 60 years and above had a lower risk of anxiety compared to younger people aged 20–39 years, and those with high educational qualifications had a lower risk of anxiety compared to those with low educational qualifications. People who were living alone had a higher risk of developing an anxiety disorder, while non-smokers had a lower risk of developing an anxiety disorder.

**Table 3:** Single factor logistic regression analysis, weighted.

Variables	Univariate	
	OR (95% CI)	*p* value
Gender		
Male	Ref	
Female	1.506(1.349,1.681)	<0.001
Age		
20–39	Ref	
40–59	1.093(0.956,1.251)	0.201
60–80	0.587(0.524,0.658)	<0.001
Race		
Mexican American	Ref	
Other Hispanic	1.402(1.109,1.774)	0.007
Non-Hispanic White	1.135(0.958,1.346)	0.150
Non-Hispanic Black	1.099(0.940,1.286)	0.243
Other Races	0.920(0.704,1.203)	0.545
Educational level		
<High school	Ref	
High school	0.753(0.650,0.871)	<0.001
>High school	0.764(0.666,0.876)	<0.001
Marital status		
Not living alone	Ref	
Living alone	1.205(1.078,1.347)	0.002
Smoking status		
Smoker	Ref	
Never smoker	0.754(0.690,0.823)	<0.001
Drinking status		
Yes	Ref	
No	0.896(0.789,1.018)	0.099
Diabetes		
Yes	Ref	
No	0.891(0.737,1.078)	0.242
Hypertension		
Yes	Ref	
No	0.930(0.824,1.049)	0.243

Table 4 shows the relationship between serum vitamin D levels and anxiety. Participants were grouped according to the interquartile range (IQR) of serum vitamin D (Q 1: 0–25%; Q 2: >25–50%; Q 3: >50–75%; Q 4: >75%) and analyzed using Q 1 as the reference group. In Model 1, no adjustment was made for any of the variables. Serum vitamin D was significantly associated with anxiety in Q 4 compared to Q 1, with an OR and 95% CI of 0.774 (0.663, 0.903). The results of Model 2, adjusted for gender, age and race, showed that serum vitamin D levels were negatively associated with anxiety with an OR and 95% CI of 0.730 (0.621, 0.857). Model 3 was adjusted for all covariates. Serum vitamin D in Q4 was significantly associated with anxiety, with an OR and 95% CI of 0.781(0.669,0.912), respectively. In all three models, *p* for trend was less than 0.001.

**Table 4:** Results of multivariate logistic analysis of the relationship between serum vitamin D and anxiety, weighted.

Serum vitamin D	Model 1	Model 2	Model 3
Q1	Ref		
Q2	0.961(0.840,1.099)	0.950(0.823,1.095)	0.982(0.859,1.123)
Q3	0.883(0.773,1.009)	0.856(0.748,0.979) *	0.894(0.781,1.023)
Q4	0.774(0.663,0.903) **	0.730(0.621,0.857) ***	0.781(0.669,0.912) **
*P* for trend	<0.001	<0.001	<0.001

*p < 0.05; **p < 0.01; ***p < 0.001. Model 1 was unadjusted, Model 2 was adjusted for gender, age, and race, and Model 3 was adjusted for gender, age, race, education, marital status, body mass index, poverty income ratio, smoking status, drinking status, hypertension, and diabetes.

**Figure 2:**
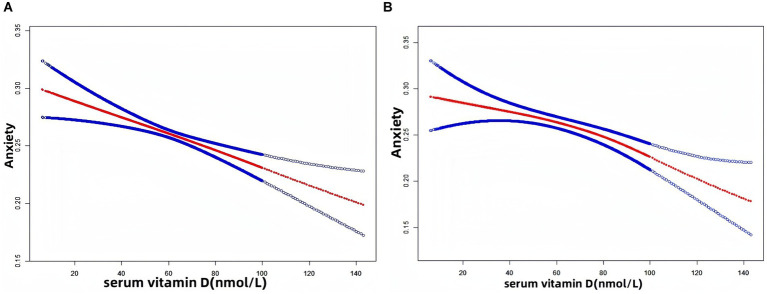
Results of smoothed curve fitting between serum vitamin D and anxiety. **(A)** Unadjusted for variables; **(B)** Adjusted for gender, age, race, education, marital status, body mass index, poverty income ratio, smoking status, drinking status, hypertension, and diabetes. The red solid arcs indicate the smoothed curve fitting between the variables. The area between the two blue dashed lines represents the 95% CI.

**Figure 3:**
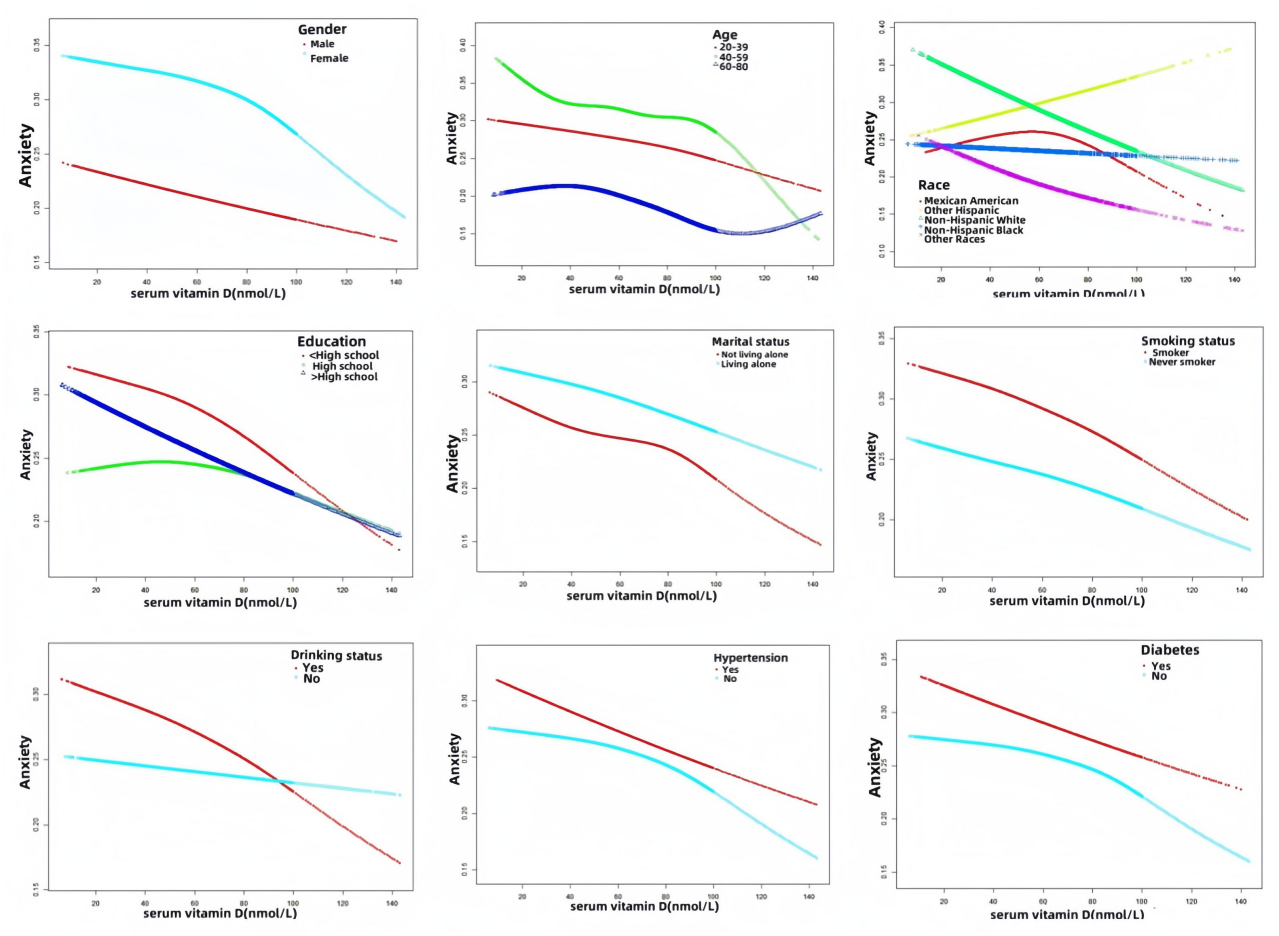
Results of smoothed curve fitting between serum vitamin D and anxiety stratified by gender, age, race, education, marital status, smoking status, drinking status, hypertension, and diabetes.

We performed a smoothed curve fit as shown in Figure 2. Figure 2A is unadjusted for variables, showing that as serum vitamin D levels increase, anxiety risk decreases. In Figure 2B, when all variables were adjusted, anxiety risk decreased as vitamin D levels increased. In Figure 3, the results of the smoothed curve fit showed an overall decreasing trend in anxiety risk with increasing serum vitamin D levels after stratifying by gender, age, race, education, marital status, smoking status, drinking status, hypertension, and diabetes, respectively.

## 4 Discussion

To our knowledge, this is the first article to use NHANES data to study the serum vitamin D-anxiety relationship. We use NHANES data of 2007–2012, and use weighted logistic regression and smoothing curve fitting analysis of serum vitamin D concentrations and the relationship between the risk of anxiety symptoms. The results showed that serum vitamin D was inversely associated with the risk of anxiety symptoms in adults.

Many studies have confirmed the beneficial effects of vitamin D on the development of anxiety disorders. A study has found that vitamin D supplements can effectively reduce the severity of generalized anxiety symptoms (18). Low vitamin D levels were linked to increased depression and anxiety symptoms in a study on the role of vitamin D in anxiety (19). In a about vitamin D supplement for older people with pre-diabetes the influence of anxiety and depression state of randomized controlled study, found that once a week for vitamin D supplement plan can effectively alleviate the high risk population of anxiety and depression symptoms (20). In addition, in a relatively young endurance athletes increase the intake of vitamin D may reduce mental anxiety (21). Finally, in a biological experiment, it was observed that mice exhibited increased anxiety-like behavior on behavioral tests after vitamin D deficiency (22).

Of course, some studies have reported results contrary to ours. There was no correlation found between depression and anxiety scores and serum vitamin D levels in a study assessing the relationship between serum vitamin D and B12 levels, nutritional levels, depression and anxiety in adult cancer patients before and after chemotherapy (13). In a study examining the relationship between vitamin D and anxiety and depression in pregnant adolescents, no relationship was found between serum vitamin D and anxiety or depression (14). In one of Australia’s young women 25—hydroxy serum vitamin D and the study of mental health, vitamin D status also proved to have nothing to do with depression or anxiety diagnosis report (23). Overall, there are inconsistent findings regarding the association between vitamin D and anxiety, which may be due to differences in demographic characteristics of the study samples, study design, environmental factors, seasonal variations, co-morbidities, and dietary habits.

The relationship between serum vitamin D and anxiety varies across demographic and lifestyle factors. Gender differences may be due to differential effects of sex hormones on vitamin D metabolism and the nervous system, whereas age differences may be related to age-related declines in the ability to synthesize vitamin D and more chronic health problems in older people. In terms of race differences, there may be genetic differences as well as social, cultural, and economic differences. Education level may reflect socioeconomic status and access to resources, while marital status may be associated with social support, family stress, and life stability. Smoking and drinking differences may relate to the effects of these habits on vitamin D metabolism and the immune system, as well as their relationship to ways of coping with stress and social activities. Finally, hypertension and diabetes differences may be due to the fact that chronic diseases indirectly affect anxiety levels by affecting the inflammatory responses (24, 25). Further research is needed in the future to confirm these findings and determine the reasons for this discrepancy.

A unique neurosteroid hormone, vitamin D’s receptors are found in neurons and glial cells in different parts of the brain, including the cingulate gyrus and hippocampus (26). Vitamin D with antioxidant and anti-inflammatory properties, promote neurogenesis and neural regulation, and adjust the gut microbiota (27). It in regulating neurotrophic factor, nerve protection, neural plasticity, brain development and neural immune adjustment plays an important role in the process of brain (28). Vitamin D deficiency may contribute to anxiety and depression (29).

Mechanisms of action between vitamin D and anxiety have been studied. Neurons and glial cells in different areas of the prefrontal cortex and hippocampus system expression of VDR and vitamin D metabolism enzymes, suggesting that vitamin D may play a role in anxiety (30). Positive regulation of antioxidant enzymes by osteotriol (a form of vitamin D) contributes to redox homeostasis and thus attenuates neuroinflammatory processes (31, 32). Vitamin D in the immune and nervous regulation part through its interaction with gut microbiota (33). Vitamin D deficiency, VDR, or CYP27B1 depletion has been shown to cause an increase in Mycobacterium anisopliae and *Mycobacterium avium*, triggering epithelial barrier dysfunction and intestinal inflammation. The positive regulation of antioxidant enzymes such as HO-1, CAT, and SOD by calcitriol contributes to the REDOX balance, thereby reducing the neuroinflammatory process (34, 35).

There are several benefits of our study as follows. To ensure the validity of our results, we used appropriate weighting and confounding adjustments in our analyses. Second, our sample size was large enough to effectively reveal the relationship between serum vitamin D and anxiety. Finally, we focused specifically on the relationship between serum vitamin D levels and anxiety. Compared to depression, relatively few studies have been conducted on the relationship between serum vitamin D and anxiety. There are also several flaws in our study. Firstly, because this was a cross-sectional study, it was not possible to determine a causal relationship between serum vitamin D and anxiety. Second, the measure of anxiety was a self-reported outcome by the subjects and may have been influenced by subjective factors. In addition, it was not possible to adjust for all covariates, and only those included were adjusted. Potential uncontrolled variables may still influence our conclusions. Finally, these data are from the American population and caution should be exercised when extrapolating to the population as a whole.

## 5 Conclusion

Serum vitamin D was inversely associated with anxiety. The future more prospective studies are needed to confirm this.

## Data availability statement

The datasets presented in this study can be found in online repositories. The names of the repository/repositories and accession number(s) can be found at: https://www.cdc.gov/nchs/nhanes.

## Ethics statement

The studies involving humans were approved by National Center for Health Statistics Institutional Review Board. The studies were conducted in accordance with the local legislation and institutional requirements. Written informed consent for participation in this study was provided by the participants’ legal guardians/next of kin. Written informed consent was obtained from the individual(s) for the publication of any potentially identifiable images or data included in this article.

## Author contributions

ZW: Conceptualization, Writing – original draft. LB: Conceptualization, Investigation, Software, Writing – original draft, Writing – review & editing. SW: Writing – review & editing. JC: Writing – review & editing. HJ: Writing – review & editing. JS: Writing – review & editing.

## Footnotes

^1^http://www.empowerstats.com
